# IGFBP in tumor immunity: a novel target for cancer immunotherapy

**DOI:** 10.1186/s12967-026-08204-z

**Published:** 2026-05-07

**Authors:** Qingqiang Lei, Liangbin Lin, Hui Yu

**Affiliations:** 1 Center of Bone Metabolism and Repair, Department of Wound Repair and Rehabilitation Medicine, State Key Laboratory of Trauma, Burns and Combined Injury, Trauma Center, Research Institute of Surgery, Daping Hospital, Army Medical University, Chongqing, 400000 China; 2 Medical Research Center, The Third People’s Hospital of Chengdu, The Affiliated Hospital of Southwest Jiaotong University, Chengdu, 610014 China; 3 Obesity and Metabolism Medicine-Engineering Integration Laboratory, Department of General Surgery, The Third People’s Hospital of Chengdu, The Affiliated Hospital of Southwest Jiaotong University, Chengdu, 610031 China; 4 The Center of Gastrointestinal and Minimally Invasive Surgery, Department of General Surgery, The Third People’s Hospital of Chengdu, The Affiliated Hospital of Southwest Jiaotong University, Chengdu, 610031 China; 5 Department of Urology, The Third People’s Hospital of Chengdu, The Affiliated Hospital of Southwest Jiaotong University, Chengdu, 610014 China

**Keywords:** Insulin-like growth factor-binding protein (IGFBP), Immune system, Tumor immunity, Cancer Immunotherapy

## Abstract

**Background:**

The insulin-like growth factor-binding protein (IGFBP) family consists of soluble bioactive molecules that, together with insulin-like growth factors (IGFs) and their receptors, are critical modulators of endocrine, metabolic, and immune functions. IGFBPs serve as signaling intermediaries in fundamental cellular processes such as migration, differentiation, proliferation, and apoptosis. Although their role in immune regulation is well-documented, this understanding has not yet led to significant clinical progress, particularly in research utilizing human specimens.

**Main body:**

This review synthesizes current knowledge on the functions and mechanisms of IGFBPs within immune regulation and tumor immunology, highlighting their therapeutic potential. We specifically examine how IGFBPs influence diverse cell populations residing in the tumor immune microenvironment, primarily through IGF-dependent and IGF-independent pathways. The article highlights future research directions and potential targets for novel immunotherapy strategies. This article also synthesizes the latest clinical research data on the correlation between IGFBP expression levels and patient prognosis across various cancer types, strengthening the translational potential of IGFBPs as targets for immunotherapy.

**Conclusion:**

By detailing the impact of IGFBPs on the tumor immune landscape, we position this protein family as promising targets for the development of novel cancer immunotherapies.

## Introduction

The immune system is a complex network composed of cells, tissues, and organs, divided into the innate system and the adaptive immune system [1]. Innate immune cells (such as macrophages, dendritic cells, and neutrophils) express various pattern recognition receptors to detect pathogens, and then produce antimicrobial factors and inflammatory mediators [2, 3]. The innate immune system can also regulate the initiation of adaptive immunity [4]. Adaptive immunity relies on T lymphocytes and B lymphocytes, both of which possess highly specific antigen recognition receptors: T cell receptors and B cell receptors [5]. B cells are responsible for producing antibodies and mediating humoral immunity, whereas T cells, including CD4^+^ and CD8^+^ T cells, are responsible for cellular immunity [6]. The regulation of the immune system is a very complex process. Insulin-like growth factors (IGFs) and their binding proteins (IGFBPs) play complex and crucial roles in the immune system, together forming a sophisticated regulatory network that affects the development, activation, function, and survival of immune cells [7–9].

The IGF family plays an important role in cell proliferation and differentiation, metabolic regulation, and physiological development [10]. IGF receptors (IGF-R) are widely expressed on various immune cells [8]. When IGF binds to its receptor, it activates downstream signaling pathways, thereby regulating the activation, proliferation, and apoptosis of immune cells [11]. IGFBP activates downstream signaling pathways through IGF and various binding proteins. The IGFBP family plays a crucial role in the immune system, extensively regulating the development, activation, function, and survival of immune cells through both IGF-dependent and IGF-independent mechanisms [12, 13]. The IGFBP family participates in the fine regulation of immune response by regulating IGF bioavailability in the immune microenvironment and directly regulating the function of immune cells independent of IGF [14].

The clinical goal of cancer immunotherapy is to prime host immune system to provide passive or active immunity against malignant tumors [15]. Tumor infiltrating immune cells play a regulatory role in tumor microenvironment which is closely related to immune escape of tumor cells, thus influence tumor progress. Tumor immune evasion results from the suppression of immune system functions, with the IGFBP family playing a pivotal regulatory role in this process. This review summarizes the latest research advancements on how IGFBP modulates tumor immune mechanisms and explores potential strategies for IGFBP-targeted immunotherapy, aiming to provide new insights for cancer prevention and treatment. However, advancing this theoretical therapeutic target to practical implementation requires further research and additional efforts. The article will also highlight future research directions and potential targets for novel immunotherapy strategies.

## The IGF system: components, signaling, and immunoregulatory roles

**Fig. 1:**
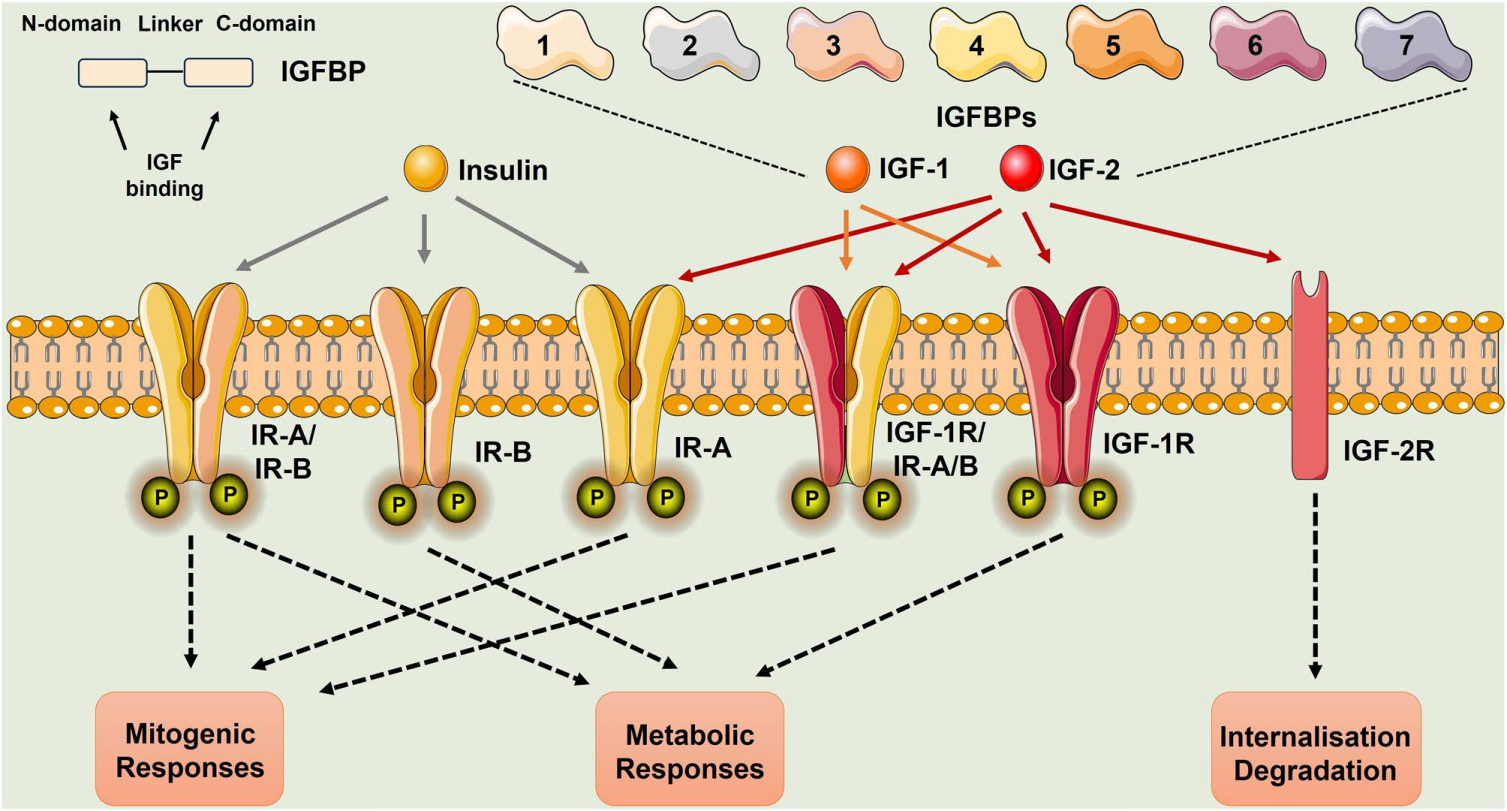
The main components of the IGF system. Three insulin peptides, IGF-1, IGF-2, and insulin, act through cell surface receptors. IGF-1 and IGF-2 regulate their availability to bind these receptors by binding with six soluble high-affinity binding proteins IGFBP-1 to IGFBP-6 and the low-affinity binding-related protein IGFBP-7. IGFBPs have three conserved subdomains: the highly conserved cysteine-rich N-terminal and C-terminal domains, and the intermediate linker domain. The N-terminal and C-terminal cysteines form disulfide bonds within the domains, keeping these terminal regions in a globular structure, which helps the high-affinity binding to IGFs. IGF-2 R also regulates the availability of IGF-2 by binding to and degrading IGF-2. Insulin has the highest binding affinity for the two insulin receptor subtypes, IR-A and IR-B. IR-A/IR-B is a hybrid receptor composed of splicing variants of the insulin receptor, which has lower binding affinity for insulin. Insulin has low affinity for IGF-1 R. Each half-receptor of IR-A, IR-B, and IGF-1 R can heterodimerize to form hybrid receptors. IGF-1 has high-affinity binding to IGF-1 R and the IGF-1 R/IR-A and IGF-1 R/IR-B hybrid receptors. IGF-II has high-affinity binding to IGF-1 R, IR-A, and IGF-IR/IR-A hybrid receptors. Activation of IR-B, IGF-1 R, and IR-A/IR-B triggers metabolic responses. Activation of IR-A, IGF-1 R, IGF-1 R/IR-A, and IR-A/IR-B triggers mitogenic responses

The IGF family includes IGF-1, IGF-2, and IGF-3 [16, 17]. IGF-1 and IGF-2 are the most common members，they play an important role in cell proliferation and differentiation, metabolic regulation, and physiological development (Fig. 1 and Table 1) [10, 25]. Although IGF-3 shares a similar tertiary protein structure with IGF-1 and IGF-2, both consisting of five domains (B, C, A, D, E), it exhibits relatively low sequence homology with them [17]. IGF-3 demonstrates stricter tissue and species specificity, being primarily expressed in the gonads of fish and playing a role in their reproduction and growth [26, 27]. There are significant differences in the physiological mechanisms between insulin and IGF, with the latter being secreted by endocrine glands and stored [28]. Early studies found that the majority of IGF molecules in the circulatory system are much larger in volume than peptide substances, as they bind to specific carrier proteins [29].

**Table 1:** Biological functions of IGF-R in signal transduction axis

Receptor	ligand	Function	References
IR-A	Insulin/IGF-2	Mitogenic Responses	[18, 19]
IR-B	Insulin	Metabolic Responses	[20]
IR-A/IR-B	Insulin	Mitogenic/Metabolic Responses	[21]
IGF-1 R	IGF-1/2	Metabolic Responses	[22]
IGF-1 R/IR-A/B	IGF1/2	Mitogenic Responses	[19, 23]
IGF-2 R	IGF-2	Degradation	[24]

There are two types of receptors in the IGF family of receptors: IGF-1 receptor (IGF-1 R) and IGF-2 receptors (IGF-2 R), all belong to the tyrosine kinase receptor family (Fig. 1 and Table 1) [30]. Insulin has the highest binding affinity for the two insulin receptor (IR) subtypes, IR-A and IR-B [18, 20]. IR-A/IR-B is a hybrid receptor composed of splicing variants of the insulin receptor, which has lower binding affinity for insulin [21]. Each half-receptor of IR-A, IR-B, and IGF-1 R can heterodimerize to form hybrid receptors [31, 32]. IGF-1 has high-affinity binding to IGF-1 R and the IGF-1 R/IR-A and IGF-1 R/IR-B hybrid receptors [22, 23]. IGF-2 has high-affinity binding to IGF-1 R, IR-A, and IGF-1 R/IR-A hybrid receptors [18, 19]. These receptors are widely distributed in various cells and tissues, includes liver, muscle, bone, brain, heart, lungs, and kidneys. It is worth noting，IGF-2 R is called an IGF receptor, however, its function is different from that of IGF-1 R [33, 34]. IGF-2 R primarily acts as a clearance receptor, recognizes and removes excess IGF-2 from the body, thus regulating the biological effects of IGF-2 [24]. Therefore, IGF-2 R also plays an important role in tumor growth, embryonic development, and metabolic regulation [35].

The IGF family and its receptors not only regulate cell growth and metabolism, but also play a crucial and complex role in the immune system. It has a wide range of regulatory effects on the development, activation, function and survival of immune cells [36–38]. The IGF family and its receptors are primarily through IGF binding to IGF-R and IGFBP Binding with IGF regulates the function of the immune system [39]. IGF-1 and IGF-2 bind to IGF-1 R and IGF-2 R，activate tyrosine kinase，then activate the PI3K/AKT, Ras/MAPK, and Wnt pathways [40]. These pathways regulate the activation, proliferation, and differentiation of immune cells. IGFBP can bind to IGF, regulates the distribution and stability of IGF in the body, and affects the biological activity and metabolism of IGF [41]. furthermore, IGFBP can also directly affect the signaling of immune cells, similar to IGF [42].

## The IGFBP family: structure, function, and regulatory roles in physiology and disease

IGFBP is a family of proteins that bind to IGF, together with IGF receptors, it regulates the biological effects of IGF [43]. IGFBP primarily affects the distribution and stability of IGF in the body, thus affecting biological activity and metabolism of IGF, and IGFBP can also directly affect cell signaling (Fig. 1 and Table 2).

**Table 2:** Biological functions of IGFBPs in signal transduction axis

Binding Protein	Source	Ligand	Function	References
IGFBP-1	Liver and Placenta	IGF-1/2	Fetal growth and metabolic stress	[44]
IGFBP-2	Liver, central nervous system, kidneys, and adipose tissue	IGF-1/2	Childhood growth, brain function, cancer	[45]
IGFBP-3	Liver, Fibroblasts, and vascular endothelial cells	IGF-1/2	Circulating IGF main repository, body axis growth	[46]
IGFBP-4	Widely distributed, expressed in many organizations	IGF-1/2	General inhibitor of local IGF activity	[47]
IGFBP-5	Kidneys, osteoblasts, mammary glands, and uterus	IGF-1/2	Bone metabolism and tissue repair	[48]
IGFBP-6	Ovaries, kidneys, and central nervous system	IGF-2	IGF-2 specific inhibitor	[49]
IGFBP-7	Endothelial cells, fibroblasts, and various epithelial cells	TGF-β	Tumor suppression, cellular senescence	[50]

The IGFBP family is a group of transport proteins, Binds to IGF and regulates its activity [51]. The subtypes of IGFBP differ in tissue expression patterns, binding affinity to IGF, and biological function [12, 52]. All IGFBP possess three conserved subdomains: highly conserved cysteine-rich N-terminal and C-terminal domains, where disulfide bonds formed by cysteines maintain these terminal regions in a rigid globular structure, both of which contribute to high-affinity IGF binding [53].

There are 6 subtypes of the IGFBP family: IGFBP-1 is mainly expressed in liver, kidney, and pancreatic cells, regulates the activity of IGF-1, inhibits cell proliferation, promotes glucose production [44]. IGFBP-2 is widely expressed in various tissues, which can promote or inhibit cell proliferation and have the effect of promoting bone growth and repair [45]. IGFBP-3 is mainly synthesized in the liver, It is the main carrier protein of IGF-1 in the blood, inhibits cell proliferation, induce apoptosis [46]. IGFBP-4 is mainly expressed in pancreatic cells, it has a significant inhibitory effect on the activity of IGF-1, it can inhibit cell proliferation, and regulates insulin secretion [47]. IGFBP-5 is widely expressed in various tissues, it can promote or inhibit cell proliferation, and is involved in the development and repair of muscles and bones [48]. IGFBP-6 is mainly expressed in the liver and kidney, inhibits the activity of IGF-1, and is involved in the metabolism and regulation of insulin-like growth factor [49].

In addition to these six classical members with high affinity for IGF, there are also IGFBP-related protein (IGFBP-rP) members with low affinity for IGF, which are structurally similar to classical IGFBP [54]. Among them, IGFBP-7 (also known as IGFBP-rP1) is the most in-depth representative member [50]. IGFBP-7, also known as angiogenesis inhibitor, has a low affinity for IGF-1 and IGF-2, but can directly bind to insulin receptors and act as an insulin antagonist, and is also considered a tumor suppressor [50, 55, 56]. Within the IGFBP superfamily, certain subtypes, such as IGFBP-3 and IGFBP-5, can enhance or inhibit IGF activity, while other subtypes, like IGFBP-4 and IGFBP-6, primarily function as inhibitors [37, 57].

Research has shown that, through IGF-dependent and IGF-independent effects, IGFBP regulates a variety of cellular processes, including proliferation, survival, migration, senescence, autophagy, and angiogenesis, and its dysregulation is associated with a range of diseases, including malignant, metabolic, neurological, and immune disorders [35, 58]. Changes in IGFBP levels may contribute to the development of these diseases and may serve as diagnostic biomarkers. For example, Elevated IGFBP-3 levels are associated with an increased risk of breast and prostate cancer, While low IGFBP-1 levels are associated with insulin resistance and type 2 diabetes [59–61]. Therefore, the IGFBP family plays a crucial role in regulating immune system function.

## The dual role of IGFBP family in tumor immunity: IGF-dependent and IGF-Independent mechanisms

The IGFBP family activates downstream signaling pathways through a variety of binding proteins such as IGF and transmembrane receptors. In both IGF-dependent and IGF-independent cell signaling pathways, the IGFBP family plays various key roles related to physiological processes such as cell proliferation and differentiation, cell death induction, and can regulate various metabolic activities such as blood glucose and lipid metabolism.

In tumor immunity, IGFBP plays an important role in both IGF-dependent and IGF-independent ways (Table 3). IGFBP indirectly affects tumor immunity by regulating IGF signaling, which is the most classic and mode of action [89]. The IGF/IGF-R signaling pathway has a broad inhibitory effect on the immune system, and IGF signaling can inhibit the activation and function of cytotoxic T cells and promote tumor immune escape [39, 62]. IGFBP inhibits its binding to IGF-R on T cells by isolating IGF, thereby indirectly lifting the inhibition of T cells and enhancing the anti-tumor immune response [63]. By inhibiting IGF signaling, IGFBP may also weaken the immunosuppressive ability of Regulatory T cells (Tregs), which is beneficial for anti-tumor immunity [90].

**Table 3:** Summary of role of IGFBP family in tumor immunity

Binding Protein	Role in tumor immunity	Types of cancer	Target cell	Mechanism	Dependent on the IGF axis	References
IGFBP-1	promote	upper gastrointestinal cancer, colorectal cancer, nasopharyngeal carcinoma, lung cancer	T cell	Indirectly relieves T cell suppression by sequestering IGF-1.	Yes	[62, 63]
IGFBP-2	suppress	epithelial ovarian cancer, metastatic prostate cancer, cervical cancer, bladder cancer, glioblastoma, endometrial cancer	M2 macrophage, tumor cell	Promote M2 macrophage polarization and PD-L1 expression in M2 macrophages. Promote VEGF and PD-L1 expression in tumor cell.	No	[64–68]
	promote	breast cancer，colorectal cancer	tumor cell	Inhibit tumor growth and reduce tumor angiogenesis.	No	[69–71]
IGFBP-3	promote	epithelial ovarian, gastric cancer, breast cancer, colon cancer, lung cancer	tumor cell	Interact with GRP78, activating caspase-7 and further inducing tumor cell apoptosis.	No	[60, 72]
IGFBP-4	promote	lung cancer, epithelial ovarian cancer, glioblastoma, metastatic hepatocellular carcinoma	tumor cell	Inhibit intracellular CatB activity and COX-2 production by colocalizing with lysosome-like structures.	No	[73, 74]
IGFBP-5	promote	breast cancer, head and neck squamous cell carcinoma, ovarian cancer, lung cancer, gastric cancer	tumor cell	Inhibit tumor vascular distribution and growth by repressing the expression of the VEGF and PIWIL1.	No	[75]
	suppress	colon cancer, pancreatic cancer, glioma	tumor cell	promotes tumor cell growth. Induce the expression of PD-L1 and CXCR4.		[76–78]
IGFBP-6	promote	colon cancer, breast cancer, nasopharyngeal cancer	tumor cell	Binds to IGF-2, preventing its interaction with IGF-1 R.	Yse	[79]
	suppress	rhabdomyosarcoma, glioblastoma	tumor cell, M2 macrophage	Binds to the transmembrane protein PHB2, activating downstream MAPK signaling pathways. Induces M2 macrophage polarization.	No	[80, 81]
IGFBP-7	promote	non-small cell lung cancer, lung cancer, breast cancer	tumor cell, T cell, DC, MDSC	Suppresses tumor growth by inhibiting IGF signaling and angiogenesis. recruiting CD8^+^ T cells to the tumor site, promotes antigen presentation by DCs, and inhibiting infiltration of MDSCs.	Yes and no	[82–86]
	suppress	colon cancer, gastric cancer,	tumor cell	Promotes the proliferation and migration of tumor cell		[87, 88].

A growing body of research shows that IGFBP can interact directly with immune cells and tumor cells without relying on IGF. Some IGFBP proteins can directly regulate macrophage polarization and T cell function. IGFBP can directly act on tumor cells, affecting tumor cell proliferation, apoptosis, and invasion. More importantly, IGFBP can regulate the expression of MHC molecules and programmed death ligand 1 (PD-L1) on the surface of tumor cells, thereby directly affecting the ease of recognition by the immune system. In addition, different members of IGFBP may have vastly different roles in tumor immunity, even the same member can play opposite roles in different cancers. IGFBP exerts complex dual effects on cancer, potentially inhibiting tumor growth or promoting tumor progression, depending on the cancer type, microenvironment, and molecular regulatory context. Understanding the specific role of IGFBP in specific cancers provides potential targets for the development of new immunotherapy strategies.

### IGFBP-1

IGFBP-1 is a protein with a molecular weight of 30 kDa, and its circulating levels are mainly produced by the liver and kidneys [91]. This protein is expressed locally in blood vessels, including vascular endothelial cells [92]. The main role of IGFBP-1 is to dynamically regulate the bioavailability of IGF in the cycle [93]. The effects of IGFBP-1 on cell migration and proliferation have been extensively studied, and its mechanism of action includes both IGF-dependent activity and IGF-independent functions [94]. The integrin-binding domain of the C-terminus of IGFBP-1 is an important mediator that mediates the independent effects of IGF [95]. Studies have shown that IGFBP-1 has a dual promoting or inhibitory effect on cancer depending on the cell type and environment [35]. Although several studies have confirmed that IGFBP-1 plays an important role in the occurrence and progression of cancer, its specific mechanism of action still needs to be further explored.

#### IGFBP-1 as potential biomarkers for cancers

Several clinical studies have shown that serum IGFBP-1 levels can be used as potential biomarkers for diagnosing a wide range of cancers. The levels of IGFBP-1 are significantly elevated in the serum of patients with upper gastrointestinal cancers [96]. Serum IGFBP-1 demonstrates high diagnostic accuracy for early-stage esophageal squamous cell carcinoma, esophagogastric junction adenocarcinoma, and gastric cancer [96]. Recent studies have found that serum IGFBP-1 can also be used as a diagnostic and prognostic biomarker for colorectal cancer [97]. IGFBP-1 is specifically expressed in ovarian clear cell adenocarcinoma and can be used as an immunohistochemical marker for ovarian clear cell adenocarcinoma [98]. A higher IGFBP-1/IGF-1 ratio in serum was significantly associated with poor prognosis in patients with nasopharyngeal carcinoma, with strong clinicopathological significance and prognostic value [99]. Elevated IGFBP-1 levels are associated with shorter castration-resistant prostate cancer time and lower overall survival in patients with metastatic prostate cancer [100]. IGFBP-1 can promote the proliferation and metastasis of gastric cancer cells, and elevated IGFBP-1 expression is associated with a decrease in overall survival in gastric cancer patients [101]. These studies show that IGFBP-1 serves as a potential diagnostic and prognostic biomarker for a variety of cancers. However, the specific molecular mechanism of IGFBP-1 needs to be further studied.

#### Function of IGFBP-1 in tumor immunity

IGF-I can protect T cells and B cells from apoptosis, while IGFBP-1 binds to IGF-I, limiting its bioavailability, thereby promoting apoptosis and depletion of T cells and B cells, and facilitating tumor development [102–104]. Recent studies have shown that in addition to being a diagnostic and prognostic marker for a variety of cancers, IGFBP-1 also has an activating effect against tumor immunity. Dietary galactose can inhibit IGF-1 signal-dependent T cell depletion and enhance CD8^+^ T cell anti-tumor response by promoting IGFBP-1 production in hepatocytes [63]. Deletion of IGF-1 R in T cells enhances anti-tumor immunity by preventing T cell depletion [62]. In addition, cancer patients with high plasma IGFBP-1 levels exhibited inhibition of T cell depletion and enhanced T cell response in tumor tissue [63]. This provides new insights for the development of more effective anti-cancer immunotherapies.

IGFBP-1 can also promote the development of resistance to tamoxifen in breast cancer cells by activating the Erk pathway, and inhibit the restoration of sensitivity of breast cancer cells to tamoxifen after inhibiting Erk phosphorylation [105]. Blood proteome analysis of lung cancer patients showed that IGFBP-1 was associated with tumor infiltration and metastasis and proliferative signaling [106]. Knockdown of IGFBP-1 in lung adenocarcinoma cells significantly inhibited its proliferation and migration, down-regulated the expression of cell cycle-related proteins, and significantly inhibited tumor burden [107]. Mechanistically, knockdown of IGFBP-1 inhibits tumor cell growth by suppressing the activation of peroxisome proliferator-activated receptor alpha (PPARα) [107].

**Fig. 2:**
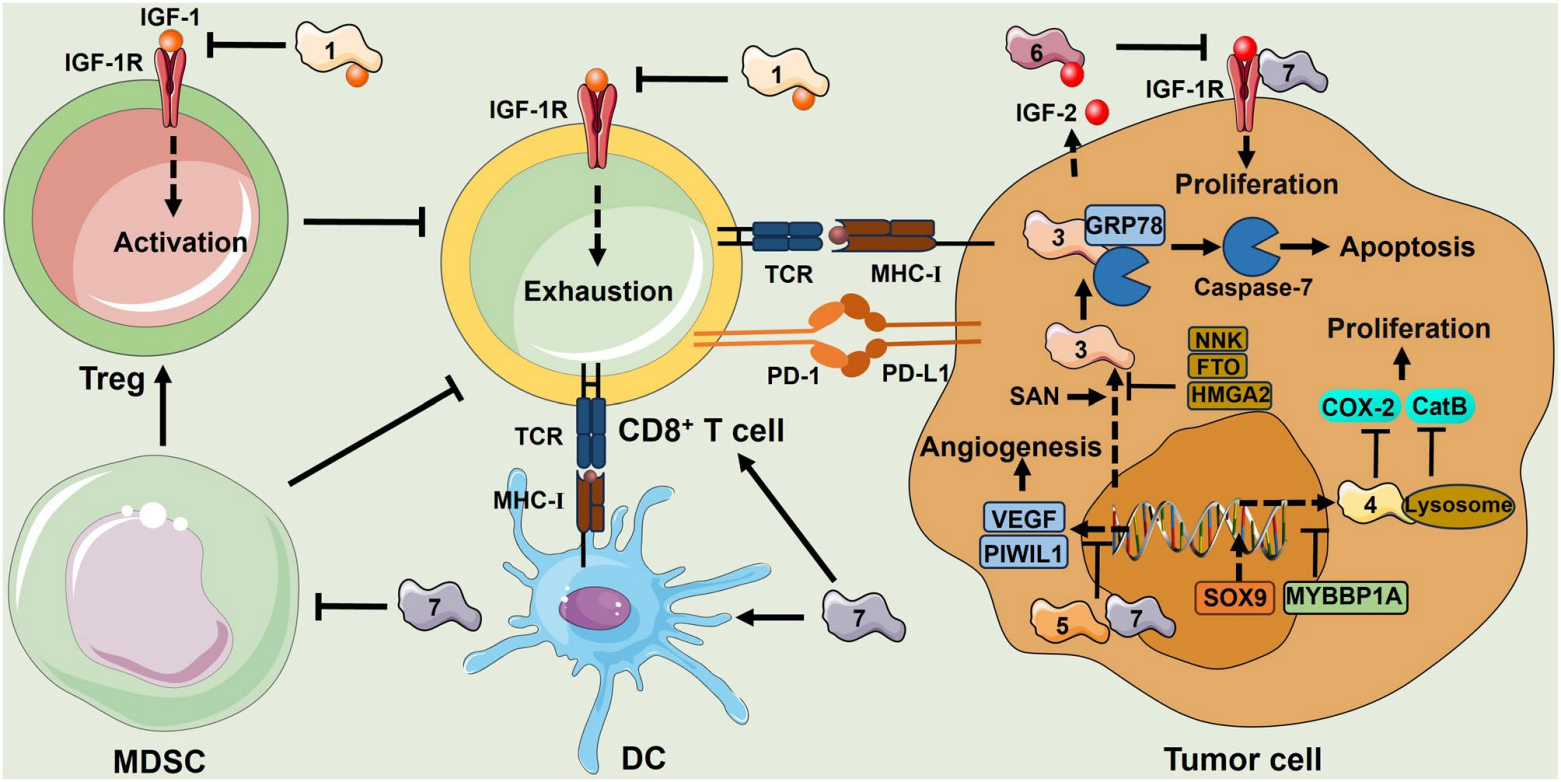
Mechanisms of the IGFBP family promotes tumor immune responses. The IGFBP family enhances tumor immune responses through IGF-dependent and IGF-independent pathways. IGFBP-1 indirectly relieves T cell suppression by sequestering IGF-1 and inhibiting its binding to IGF-1 R on CD8^+^ T cells, thereby enhancing anti-tumor immune responses. By inhibiting IGF signaling, IGFBP-1 also weakens the immunosuppressive capacity of tregs, favoring anti-tumor immunity. IGFBP-3 interacts with glucose-regulated protein (GRP78), leading to the disruption of the GRP78-caspase-7 complex, thereby activating caspase-7 and further inducing tumor cell apoptosis. Sanguinarine (SAN) can promote apoptosis in tumor cells by upregulating the expression of IGFBP-3. Nicotine-derived nitrosamines (NNK), obesity-associated protein (FTO), and chromatin-structuring protein (HMGA2) promote tumor cell growth by inhibiting IGFBP3 transcription. IGFBP-4 inhibits intracellular cathepsin B (CatB) activity and cyclooxygenase 2 (COX-2) production by colocalizing with lysosome-like structures, thereby suppressing tumor cell proliferation. Activation of the transcription factor SOX9 promotes IGFBP-4 transcription, while MYB-binding protein 1A (MYBBP1A) inhibits its transcription. IGFBP-5 suppresses angiogenesis by regulating the VEGF signaling pathway and inhibits tumor vascular distribution and growth by repressing the expression of the stem cell self-renewal protein (PIWIL1). IGFBP-6 binds to endogenously produced IGF-2, preventing its interaction with IGF-1 R, thereby inhibiting tumor cell proliferation. IGFBP-7 suppresses tumor growth by inhibiting IGF signaling and angiogenesis. IGFBP-7 can act as a chemokine, specifically recruiting CD8^+^ T cells to tumor sites. In addition, IGFBP-7 promotes antigen presentation by dendritic cells (DCs) and inhibiting infiltration of myeloid-derived suppressor cells (MDSCs), thereby enhancing the tumor immune response

At the same time, some studies have found that Helicobacter pylori can induce the expression of IGFBP-1 in gastric cancer cells, inhibit the migration of gastric cancer cells, and play a protective role in the occurrence of gastric cancer, but the specific molecular mechanism is unknown [108]. Hepatic spart-like transcription factor 1 (SALL1) in hepatocellular carcinoma cells can inhibit the proliferation of human hepatocellular carcinoma cells by promoting IGFBP-1 expression, thereby inhibiting the development of hepatocellular carcinoma [109]. These findings suggest that IGFBP-1 may act as a tumor suppressor in a variety of cancers and contribute to the development of new therapeutic strategies (Fig. 2).

### IGFBP-2

IGFBP-2 is a protein with a molecular weight of 31.4 kDa and is the second most abundant insulin-like growth factor-binding protein in the circulatory system [110]. Similar to IGFBP-1, it contains the RGD integrin-binding domain at the C-terminus, as well as the heparin-binding domain (HBD) and the nuclear localization sequence (NLS), which are responsible for promoting extracellular matrix (ECM) binding and nuclear localization, respectively [95, 111]. IGFBP-2 is involved in the regulation of IGF activity in the nervous system, peripheral tissues, and organs [112]. In addition to binding to IGF in circulation, these IGF-regulatory activities of IGFBP-2 involve interactions with extracellular matrix components and cell surface proteoglycan and integrin receptors. IGFBP-2 plays an important role in development, metabolism, and malignancy [113].

IGFBP-2 is highly expressed in both serum and tumor tissue of most cancer patients and is considered one of the most important genes in the main cancer signature. IGFBP-2 primarily regulates circulating IGF levels, however, there is substantial evidence that IGFBP-2 may also act independently of IGF. These IGF-independent effects of IGFBP-2 are exerted either through cell surface interactions or intracellular through interactions with cytoplasm or nucleus.

#### The promoting tumor role and clinical significance of IGFBP-2

**Fig. 3:**
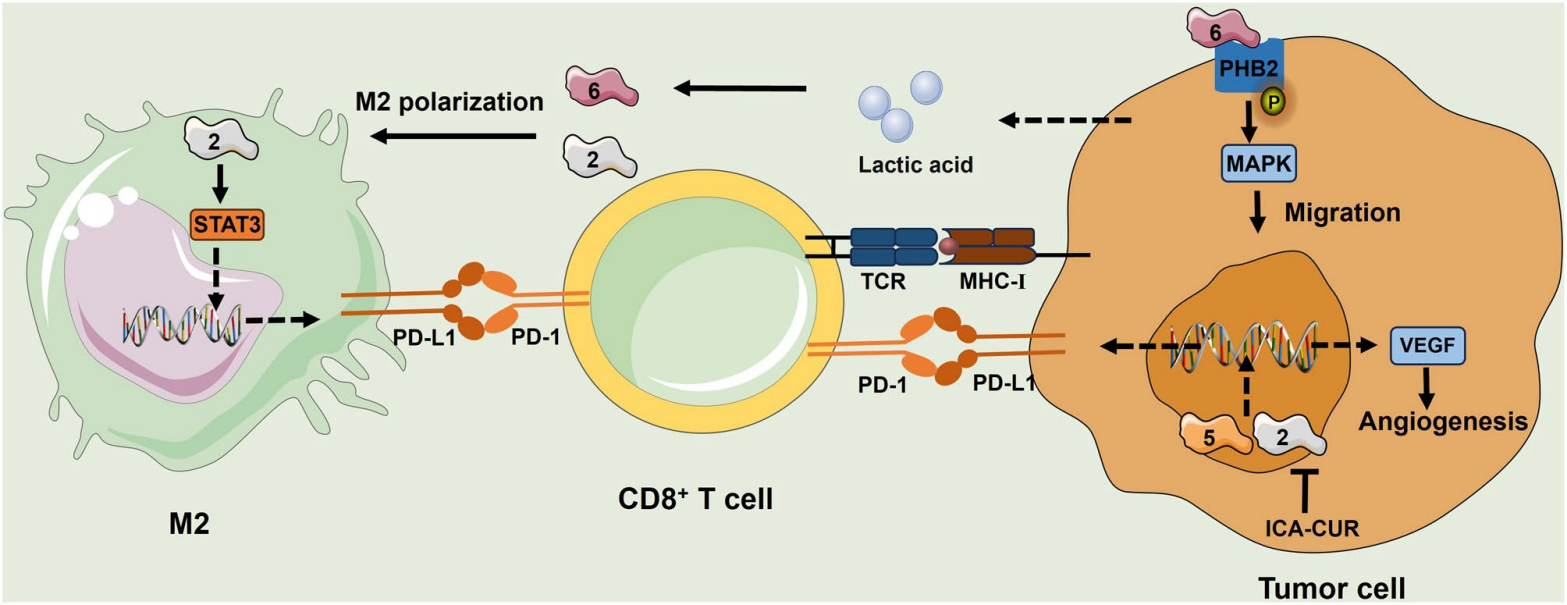
Mechanisms of the IGFBP family suppresses tumor immune responses. The IGFBP family mainly suppresses tumor immune responses through IGF-independent mechanisms. Among them, IGFBP-5 and IGFBP-6 have dual roles in promoting and inhibiting tumor immunity. IGFBP-2 can promote M2 macrophage polarization in the tumor microenvironment and activate signal transducer and activator of transcription 3 (STAT3) to enhance programmed death ligand 1 (PD-L1) expression in M2 macrophages, thereby inhibiting CD8^+^ T cell proliferation and activation. IGFBP-2 possesses a functional nuclear localization signal (NLS) sequence, which can promote VEGF and PD-L1 expression via nuclear translocation, exerting a pro-tumor effect. The combination therapy of icaritin and curcumin (ICA-CUR) can inhibit the tumor cell IGFBP2/PD-L1 pathway, thereby activating CD8^+^ T cells. IGFBP-5 can also induce PD-L1 expression in the tumor microenvironment, suppressing tumor immunity. The C-terminal domain of IGFBP-6 can bind to the prohibitin-2 (PHB2), increase PHB2 tyrosine phosphorylation, activate downstream MAPK signaling pathways, and promote tumor cell migration. Furthermore, in the tumor microenvironment, tumor cells can promote IGFBP-6 expression by producing lactate, which in turn induces M2 macrophage polarization and promotes tumor growth

IGFBP-2 promotes tumor progression across various cancers by enhancing proliferation, metastasis, angiogenesis, and immunosuppression via multiple pathways, positioning it as a valuable prognostic biomarker and therapeutic target (Fig. 3).

Patients with epithelial ovarian cancer and metastatic prostate cancer had significant increases in serum IGFBP-2, and with highly sensitive serum tumors marker Cancer Antigen 125 (CA 125) and serum prostate-specific antigen (PSA) positively correlated [114, 115]. Therefore, alterations in serum IGFBP-2 levels may serve as potential additional markers for ovarian and prostate cancer. The expression level of IGFBP-2 in gastric cancer tissues was significantly higher than that in normal mucosal and peritoneal metastases. It suggests that IGFBP-2 expression may play an important role in the proliferation, differentiation and peritoneal metastasis of gastric cancer, which may provide a reference for the clinical treatment and prognosis of gastric cancer. In cervical cancer, IGFBP-2 exerts oncogenic effects through non-IGF mechanisms, manifested by high expression of IGFBP-2 in cervical cancer cells, while IGF-1 and IGF-1 R gene expression are down-regulated [116]. Elevated IGFBP-2 is closely related to oral cancer metastasis and progression, affecting not only migration and invasion, but also wound healing ability and cell proliferation [117]. It suggests that IGFBP-2 has the potential to be used as a prognostic biomarker for oral cancer and an innovative target for the treatment of tumor invasion. Inhibition of IGFBP-2 expression in bladder cancer cells increased the sensitivity of bladder cancer cells to cisplatin [118].

IGFBP-2 has a functional nuclear localization signal (NLS) sequence, which can actively translocate into the nucleus through the classical nuclear input mechanism, activate vascular endothelial growth factor (VEGF) expression and promote angiogenesis, and play a tumor-promoting role [64]. IGFBP was highly expressed in the tumor microenvironment of low-grade glioma and malignant glioblastoma-2, which binds to the extracellular matrix to enhance the proliferation and metastasis of neuroblastoma cells, which is mediated by its HBD [111]. Further studies have shown that IGFBP-2 can both increase VEGF expression and promote angiogenesis [65, 66], and can induce M2 macrophage polarization to promote glioma progression [67]. In the tumor microenvironment, IGFBP2 induces M2 polarization of macrophages in pancreatic ductal adenocarcinoma through the signal transducers and transcriptional activator 3 (STAT3) pathway, thereby promoting tumor progression [119]. In cell co-culture experiments, it was found that human mature adipocytes increased the invasion ability of human breast cancer cells (MCF-7) by secreting IGFBP-2 [120]. In vivo, IGFBP-2 levels are higher in adipocytes surrounding metastatic breast tumors than in adipocytes surrounding non-metastatic breast tumors [120]. In addition, breast cancer cells can also enhance their own proliferative potential by producing IGFBP-2 and protect them from chemotherapy-induced death [121].

In vivo mouse tumor model studies, it was found that IGFBP-2 promotes the expression of PD-L1 in M2-like macrophages by activating STAT3, inhibits the proliferation and activation of CD8^+^ T cells, and exerts immunosuppressive effects in a PD-L1-dependent manner [68]. This may be a potential immune checkpoint inhibitor in clinical studies treatment is not efficacious, IGFBP-2 may be a target for ICI combination therapy. IGFBP-2 is highly expressed in endometrial cancer tissues and is associated with poor prognosis, with overexpression of IGFBP-2 promoting proliferation and glycolysis of endometrial cancer cells, while knockdown of IGFBP-2 has the opposite effect [122]. Mechanistically, IGFBP-2 directly interacts with PKM2 to promote endometrial cancer tumor growth through the IGFBP2/PKM2/HIF-1α axis [122]. Therefore, IGFBP2 is an upstream activator of PKM2-driven proliferation and glycolysis in endometrial cancer cells, which provides a new target for its treatment.

#### The tumor-suppressing effect of IGFBP-2

While most studies have shown that IGFBP-2 is expressed increased in a variety of cancers and can be used as a cancer biomarker. However, some studies have reported that IGFBP-2 can also inhibit tumor growth (Fig. 2). Early detection of IGFBP-2 promotes apoptosis in the human breast cancer cell line Hs578T, which is independent of IGF [69]. In mouse models, IGFBP-2 overexpression was found to reduce tumor growth by inhibiting reduced tumor cell proliferation and the appearance of abnormal crypt lesions before and during colorectal cancer [70]. IGFBP-2 can effectively inhibit tumor growth and reduce tumor angiogenesis in mouse models of breast cancer after removing protease cleavage and modification of matrix binding sites [71]. These two modifications are designed to block IGFBP-2 from combining with both IGF binding to evaluate the independent efficacy of IGFBP-2 proteins.

### IGFBP-3

IGFBP-3 is a protein with a molecular weight of 28.7 kDa, which is synthesized mainly in the liver and is the main carrier of IGF in circulation. Among the six high-affinity IGFBPs of IGF, IGFBP-3 is the most abundant IGF-binding protein in human serum, a growth-inhibiting, apoptosis-inducing molecule that has been heavily studied in cancer, and its IGF/IGF-IR-IR-independent biological role extends beyond its role in regulating IGF in cancer [123–126]. IGFBP-3 is abnormally expressed in a variety of tumor types, and its serum and tumor tissue levels provide auxiliary information for assessing tumor malignancy and patient prognosis, making it a potential therapeutic target for human malignant tumors and has significant clinical value for determining patient prognosis [127–129]. Over the past two decades, substantial evidence has revealed tumor suppressive and tumor-promoting effects of IGFBP-3, depending on cell type, post-translational modifications, and assay methods.

#### The tumor suppressive effect of IGFBP-3

Unlike IGFBP-2, IGFBP-3 has intrinsic biological activities such as anti-tumor, and is more used as a tumor suppressor to inhibit tumor growth. The level of IGFBP-3 in serum was reduced in patients with epithelial ovarian, gastric and breast cancer, suggesting that IGFBP-3 may play a role in inhibiting tumor growth (Fig. 2) [59, 114, 128]. Mechanistic studies have shown that IGFBP-3 interacts with glucose regulatory protein (GRP78) in breast cancer cells, leading to the destruction of the GRP78-caspase-7 complex, thereby activating caspase-7 and further inducing apoptosis in drug-resistant breast cancer cells, enhancing estrogen-resistant breast cancer cell pairs Fulvestrant. [60, 72]. Fulvestrant is an antiestrogen drug that works not only by blocking estrogen receptors, but also by reducing the number of estrogen receptors for breast cancer treatment [130, 131]. Some researchers have also found that DNA repair targeting IGFBP-3 may enhance the chemosensitivity of triple-negative breast cancer, thereby improving patient outcomes [132].

Similarly, serum IGFBP-3 levels in colon cancer patients were lower than those in the control group, and the early stage of cancer (I and II) serum IGFBP-3 levels were higher than those in advanced stage (III and IV) [133]. Therefore, serum IGFBP-3 may be a potential biomarker for colon cancer. Meta-analysis shows thatIGFBP-3 levels in lung and esophageal cancer patients are inversely correlated with lung cancer risk [129, 134, 135]. Suggests that low IGFBP-3 levels are associated with high cancer risk, poor prognosis, and adverse tumor staging and metastasis. Research has found that sanguinarine (SAN) can promote apoptosis in liver cancer cells by upregulating the expression of IGFBP-3, revealing a new mechanism in which IGFBP-3 is an important target of SAN [136]. Nicotine-derived nitrosamine ketone (NNK) is the most potent tobacco carcinogen and can enhance cell proliferation in lung epithelial cells [137, 138]. NNK simultaneously inhibits the expression of IGFBP-3 through DNA methylation, thereby eliminating the antitumor effect of the IGFBP-3/IGFBP-3 R system in smoking-related lung cancer [139]. Obesity-associated protein (FTO) inhibits the translation of IGFBP-3 by mediating the demethylation of m6A sites in the 3’ untranslated region of IGFBP-3 mRNA, thereby regulating the activation of the AKT signaling pathway and promoting the development of lung cancer [140]. Another study reported that T cell-specific deletion of FTO led to apoptosis of activated CD8^+^ T cells, which may be mediated by IGFBP-3 [141]. This is understanding post-transcriptional regulation of CD8^+^ T-cell immune function and potential therapeutic interventions provide new insights.

#### The carcinogenic effect of IGFBP-3 and its complex regulation

In glioma, the chromatin structural protein HMGA2 is regulated by inhibiting IGFBP3 transcription and regulation The PI3K/AKT pathway promotes the glioma malignant phenotype [142]. In pancreatic cancer, tumor cells express semaphorin7A (SEMA7A), a GPI-anchored glycoprotein with neuroimmune functions and a key regulator in inflammatory diseases and tumor development [143–145]. SEMA7A binds to tumor-associated fibroblasts and induced IGFBP-3 secretion, and IGFBP-3 promoted the expression of IL-17RB in tumor cells.IL-17B/IL-17RB pathway promotes pancreatic cancer invasion and metastasis [146, 147]. This underscores the importance of local tumor cell and fibroblast interactions in promoting cancer cell aggressiveness and provides a basis for the development of new therapeutic strategies for this communication. In a mouse breast cancer model, IGFBP-3 was found to promote breast tumor growth, and the mechanism may be to inhibit the accumulation of CD8^+^ T cells in tumor tissue, and the specific molecular mechanism still needs to be further explored [148]. In ovarian cancer cells, NF-κB binds to the promoter of miR-19a-3p and inhibits the expression of IGFBP-3, thereby promoting the proliferation and migration of ovarian cancer cells in vitro and promoting tumor growth in vivo [149]. It suggests that the influence of NF-κB/miR-19a-3p/IGFBP-3 pathway on ovarian cancer may contribute to the development of new diagnosis and treatment of ovarian cancer.

In patients with primary prostate cancer, the high nuclear localization of IGFBP-3 is associated with poor prognosis [61]. Therefore, nuclear localization of IGFBP-3 can be used as a potential new prognostic marker in prostate cancer. While the value of circulating IGFBP-3 levels as a predictor of prostate cancer remains controversial, IGFBP-3 hyper nuclear localization is one of the strongest predictors of cancer recurrence in patients with low-grade prostate cancer and may play an important role in risk stratification. Overall, IGFBP-3 is an important regulator in mediating tumorigenesis and progression, and a deeper understanding of the basic characteristics of IGFBP-3 and its role in various tumor types will provide new strategies for developing cancer diagnosis, treatment, and prognostic assessment.

### IGFBP-4

IGFBP-4 is a 24 kDa protein produced by the liver and is currently the smallest known IGFBP [150]. Unlike other IGFBPs, IGFBP-4 inhibits the effects of IGF-I and IGF-II in almost all cellular environments [151]. IGFBP-4 has three domains, where the N-terminal sequence is important for IGF binding, which acts as a transporter for IGF-I and IGF-II and regulates their biological effects [150]. There is growing evidence that IGFBP-4 inhibits IGF-induced cell growth both in vitro and in vivo, and IGFBP-4 can also mediate its effects through mechanisms independent of IGF [152]. IGFBP-4 levels and expression are influenced by IGFBP proteases, nutrition, multiple growth factors, and hormones in various tissues. Several studies have shown that regulating the inhibitory effect of IGFBP-4 on IGF-I can reduce angiogenesis and inhibit IGF’s induction of Treg cells, thereby limiting tumor development, and the expression level of IGFBP-4 in most cancers is related to its differentiation status (Fig. 2) [73, 153, 154].

#### The role and mechanism of IGFBP-4 in various cancers

The study found that IGFBP-4 was negatively correlated with the prognosis of lung cancer patients, and the serum level of IGFBP-4 in lung cancer patients was increased, and IGFBP-4 was knocked down to inhibit the growth of lung cancer cells [155, 156]. IGFBP-4 can also reduce cyclooxygenase by inhibiting the phosphorylation of PI3K/AKT, ERK, and CREB, it inhibits the proliferation, migration and invasion of lung cancer cells, and exerts an effective anti-tumor effect in non-small cell lung cancer cells [157]. IGFBP-4 can be used as a biomarker for lung cancer, it provides a potential target for lung cancer diagnosis and treatment.

In patients with epithelial ovarian cancer, serum levels of IGFBP-4 were elevated at all stages of epithelial ovarian cancer, and RNA sequencing of tumor tissue showed high expression of IGFBP-4, and malignant diseases were compared with benign diseases.IGFBP-4 levels were significantly increased [158]. IGFBP-4 expression is significantly reduced in primary glioblastoma and higher in metastatic glioblastoma [74]. Studies have demonstrated that the IGFBP-4 C-terminal protein fragment (CIBP-4) contains the thyroglobulin type 1 (Tg1) domain, and proteins with the Tg1 domain have been shown to inhibit cathepsin, a lysosomal enzyme involved in basement membrane degradation and involved in tumor invasion and angiogenesis [73, 74]. CIBP-4 can colocalize with lysosome-like structures in glioblastoma cells, inhibiting growth factor-induced intracellular cathepsin B (CatB) activity, thereby inhibiting tumor growth in animal models of glioblastoma [73]. The pleiotropic anti-angiogenic and anti-tumoriogenic activity of CIBP-4 is likely the basis for IGFBP-4‘s therapeutic potential against cancer.

SOX9 is a transcriptional regulator of IGFBP-4 in colonic epithelial cells, and SOX9-induced activation of IGFBP-4 may be one of the mechanisms by which SOX9 inhibits cell proliferation and colon cancer progression [159]. Clinical data indicate that in patients with metastatic hepatocellular carcinoma, low expression of IGFBP-4 and high expression of MYB binding protein 1A (MYBBP1A), especially in patients with lymphatic metastasis, are associated with lower patient survival rates [160]. MYBBP1A is a nucleolar transcription regulator, identified by its ability to specifically bind to the MYB oncogene protein, and possesses potential tumor suppressor activity [161–163]. Mechanism research finds, IGFBP-4 Downregulation in metastatic hepatocellular carcinoma is mediated by methylation-dependent degradation of the IGFBP-4 promoter region targeted by MYBBP1A [160]. This reveals the importance of the MYBBP1A/IGFBP-4 pathway in the progression and metastasis of metastatic hepatocellular carcinoma.

#### The regulation and therapeutic potential of IGFBP-4

IGFBP-4 was down-regulated in serum in cervical cancer patients, it is helpful for cervical cancer diagnosis and is an independent risk factor for poor prognosis in cervical cancer patients. In intrahepatic cholangiocarcinoma, miR-122-5p promotes its mRNA transcription by binding to the IGFBP-4 promoter region, inhibiting tumor invasion and metastasis in a mouse in situ metastasis model [164]. Bone marrow mesenchymal stem cell conditioned medium (MSC-CM) can inhibit the in vitro growth of several human tumor cell lines and in vivo growth of mouse squamous cell carcinoma cell lines, and reduce neovascularization [165]. Further studies have found that MSC-CM inhibits tumor growth and neovascularization in an IGFBP-4-dependent manner, suggesting that cell-free therapy using MSC-CM may be a safer and promising alternative for cancer patients [165].

N6-methyladenosine (m6A) mRNA methylation is thought to be a gene regulatory mechanism involved in disease progression and carcinogenesis [166]. Methylation of m6A mRNA in endometrial cancer cells promotes tumor formation in vivo, by reducing IGFBP-4 expression and inhibiting ERK, AKT, and NF-κB pathways, thereby promoting malignant proliferation of cancer cells and tumor formation [167]. These studies show that IGFBP-4 acts as a biomarker for a variety of cancers, It provides potential targets for its diagnosis and treatment.

### IGFBP-5

IGFBP-5 is a protein with a molecular weight of 28.6 kDa, which binds to IGF in a similar way to IGFBP-3 [28]. When IGFBP-5 binds to extracellular matrix proteins, its affinity for IGF decreases, thereby releasing IGF from the ternary complex formed by IGFBP-5 with IGF and acid unstable subunits [168]. Through this mediating effect, IGFBP-5 is able to modulate the effects of IGF, but it has also been found to have a mechanism of action independent of IGF. IGFBP-5 plays a role in cell growth, differentiation, and apoptosis. IGFBP-5 is abnormally expressed in various tumors and also plays a dual role in inhibiting and promoting cancer, depending on the type of tumor cells (Fig. 2).

#### The tumor suppressive effect of IGFBP-5

In tumor tissues of breast cancer patients, IGFBP-5 mRNA expression levels are reduced, which is opposite to IGFBP-4 expression, which is associated with the prognosis of breast cancer patients [169]. Further studies have found that IGFBP-5 can enhance TNF-α-induced growth inhibition in human breast cancer cells, while IGFBP-3 has the opposite effect [170]. It is shown that although IGFBP-3 and IGFBP-5 have many structural and functional homologies, they can regulate different apoptotic pathways in human breast cancer cells. IGFBP-5 and CCAAT/enhancer-binding protein α (C/EBPα) are both inhibitors of head and neck cancers and are downregulated in oral squamous cell carcinoma [171]. At the same time, studies have shown that IGFBP-5 has the function of inhibiting tumorigenesis of head and neck squamous cell carcinoma independent of IGF, which is of great value for future tumor intervention [172].

In human ovarian cancer xenograft models, IGFBP-5 can inhibit tumor growth and inhibit tumor vascularization, indicating that IGFBP-5 can play a tumor suppressive role by inhibiting angiogenesis [173]. Further studies found that the C-terminus of IGFBP-5 inhibits angiogenesis by regulating AKT/ERK and NF-κB-VEGF/MMP-9 signaling pathways, and exerts anti-cancer activity in ovarian cancer [174].

RASSF1C is the main isoform of the ras associated domain family member 1 (RASSF1) gene and functions as an oncogene [175–177]. In lung cancer cells, RASSF1C can promote the proliferation of lung cancer cells by up-regulating the expression of the stem cell self-renewal protein PIWIL1 [75, 178]. Further studies have found that the interaction of IGFBP-5 with RASSF1C can prevent RASSF1C from upregulating the expression of PIWIL1, thereby inhibiting lung cancer cell growth [179].

The expression of miR-103a-3p in gastric cancer tissues was significantly increased compared with neighboring non-cancerous tissues, and the upregulation of miR-103a-3p helped enhance the proliferation, invasion and migration of gastric cancer cells [180]. Mechanistic studies have shown that miR-103a-3p can directly target IGFBP-5 in gastric cancer, and IGFBP-5 overexpression can attenuate the progression induced by miR-103a-3p in gastric cancer cells [180]. Suggesting that the miR-103a-3p/IGFBP5 axis may play a role in gastric cancer progression, highlighting its potential as a therapeutic target and prognostic marker.

#### The tumor-promoting effect of IGFBP-5

In addition to exerting tumor-inhibiting activity, IGFBP-5 also has the function of partially promoting tumorigenesis (Fig. 3). Studies have found that ten-eleven translocation (TET) can inactivate Wnt/β-catenin signaling by down-regulating the expression of IGFBP-5, it exerts anti-cancer activity in colon cancer cells [76]. In pancreatic cancer cells, IGFBP-5 expression promotes cell growth by affecting cell cycle and survival signaling pathways [77].

IGFBP in malignant gliomas-5. The level is significantly increased, it can promote the proliferation, migration and invasion of glioma cells [78]. Further studies have shown that IGFBP-5 can induce the expression of PD-L1 and CXCR4 in the glioma microenvironment [78]. It suggests that IGFBP-5 as an oncogene is a biomarker of glioma prognosis. Diffuse infiltration is the main cause of drug resistance and recurrence of glioblastoma [181]. It was found that IGFBP-5 binds to the receptor tyrosine kinase 1 (ROR1) as a ligand, promotes the formation of ROR1/human epidermal growth factor receptor 2 (HER2) heterodimer and enhances the oncogenic signaling of cAMP response element-binding protein (CREB), thereby promoting glioblastoma invasion and development [182].

In addition, lentivirus-mediated IGFBP-5 knockdown and nanocapsule-mediated Cas9/sgIGFBP-5 delivery significantly reduced tumor invasion and prolonged the survival of in situ tumor-bearing mice [182]. Keloid (KD) are non-cancerous fibroproliferative tumors that exhibit cancer-like features, including uncontrolled aggressive growth, no spontaneous regression, and significantly higher recurrence rates [183]. The latest study found that Schwann cells can infiltrate and secrete IGFBP-5 to promote KD growth. Targeting IGFBP-5 or Schwann cell infiltration may provide a new therapeutic strategy for KD [184]. These studies reveal the key role of IGFBP5 in enhancing tumor invasion and development and offer a possible therapeutic approach for a variety of cancers as a potential therapeutic target.

### IGFBP-6

IGFBP-6 is a 22.8 kDa protein produced in the liver and has a 50-fold higher affinity for IGF-II than IGF-I [185]. The main role of IGFBP-6 is to inhibit IGF-II functions, such as cell proliferation, migration, and differentiation, but it has also been reported to have IGF-independent effects [186]. IGFBP-6 is involved in a variety of cellular processes and is an important factor in the immune response. It also plays a very important role in tumor biology [187, 188]. A major challenge in the future is to explore the relative effects of IGF-dependent and non-dependent IGFBP-6, which may lead to the development of treatments for diseases, including cancer.

#### The tumor suppressive effect of IGFBP-6

In colon cancer, IGFBP-6 prevents IGF-2 from interacting with IGF-1 R through autocrine mechanisms by binding to endogenously produced IGF-2, thereby inhibiting colon cancer cell invasion and migration by inhibiting cell proliferative activity and cell cycle [79, 189].

In patients with progesterone receptor-positive breast cancer, high levels of IGFBP-6 can improve overall survival [190]. IGFBP-6 is transcriptionally induced by progesterone in T47D breast cancer cells, resulting in an increase in intracellular and extracellular IGFBP-6 protein [190]. It has been shown that IGFBP-6 is a modulator of progesterone action and plays a protective role in breast cancer. In addition, IGFBP-6 was inversely correlated with the expression of most glucose metabolism-related genes, which may inhibit the energy metabolism of cancer cells through glucose metabolism-related pathways [191].

IBFBP-6 expression in nasopharyngeal carcinoma cell lines and immunolocalization in nasopharyngeal carcinoma tissues were very low [192]. Further research found thatIGFBP-6 binds to the EGR-1 promoter region and activates its expression, thereby inhibiting the progression of nasopharyngeal cancer [192].

#### The tumor-promoting effect of IGFBP-6

IGFBP-6 usually inhibits the proliferation and invasion of IGF-2-dependent cancer cells by directly inhibiting IGF-2 effects. However, in some cases, IGFBP-6 is associated with increased tumorigenicity of cancer cells, which may be played through non-IGF-2 dependent pathways (Fig. 3). The precise molecular mechanisms of IGFBP-6‘s non-IGF-independent effects are largely unknown.

In rhabdomyosarcoma cells, the C-terminal domain of IGFBP-6 can bind to the prohibitin2 (PHB2), increasing the tyrosine phosphorylation of PHB2 on the rhabdomyosarcoma cell membrane, activating the downstream MAPK signaling pathway, and promoting the migration of rhabdomyosarcoma cells [80, 193]. In the tumor microenvironment of glioblastoma, the production of lactate by glioblastoma cells promotes the expression of IGFBP-6, which in turn leads to an increase in M2 markers and a decrease in inducible nitric oxide synthase (iNOS) levels in microglia, promoting tumor growth [81]. This indicates that lactate/IGFBP-6 metabolism can reshape the tumor microenvironment in glioblastoma and activate immune escape. Therefore, in addition to inhibiting tumor cell proliferation by inhibiting IGF-2, IGFBP-6 also promotes its proliferation and migration through unique pathways, and understanding these different roles of IGFBP-6 can provide reference for the development of novel cancer therapies.

### IGFBP-7

IGFBP-7 is the most well-studied member of the IGFBP-rP protein subfamily, which not only binds IGF with low affinity, but also directly binds to and inhibits the insulin receptor itself with high affinity, thus playing a key role in cell proliferation, senescence, and apoptosis [56, 194]. IGFBP-7 (also known as mac25) was originally cloned from leptomeningeal cells, its amino acid sequence is highly similar to that of other human IGFBP proteins, conforms to the structural characteristics of IGFBP family members, and can specifically bind to IGF-I and IGF-II, but has a lower affinity than other IGFBP family members [195]. In addition, IGFBP-7 can also bind to unbound IGF-1 receptors and regulate protein synthesis, cell proliferation, and activation by inhibiting downstream signaling [196]. Unlike the complexity of the role of classical IGFBP in cancer, IGFBP-7 is generally considered a tumor suppressor gene. IGFBP-7 is widely distributed in normal tissues but is less expressed in a variety of cancer cells, suggesting that IGFBP-7 may play a role as a growth inhibitor (Fig. 2) [195]. IGFBP-7, along with high-affinity IGFBP1-6, constitutes an IGFBP superfamily that functions in IGF-dependent or IGF-independent modes to regulate normal and tumor cell growth [54].

#### IGFBP-7 acts as a tumor suppressor factor

IGFBP-7 is a low affinity IGFBP that functions more in tumor cells in an IGF independent manner. The expression of IGF-1 in the serum and lung cancer tissue of non-small cell lung cancer patients was significantly higher than that in the control group, while the expression of IGFBP-3 and IGFBP-7 was significantly lower than that in the control group [197]. Suggests that IGF-1 upregulation and IGFBP-3 and IGFBP-7 downregulation may be potential diagnostic biomarkers for non-small cell lung cancer. Recent studies have shown the critical role of IGFBP-7 in lung cancer risk assessment, early detection, prognosis assessment, and drug resistance assessment [198]. Serum IGFBP in gastric cancer patients-7 were lower than those in the normal control group, Indicates serum IGFBP-7 May be used as a potential early diagnostic marker for gastric cancer [199]. Other studies have shown that it can induce apoptosis of M12 prostate cancer cells [200].

Mechanistic studies have shown that IGFBP-7 can change epithelial mesenchymal transition marker genes through AKT/GSK3β/β-catenin signaling and inhibit the invasion of squamous carcinoma cells in the head and neck [201]. STAT5a, as a very important transcription factor, has been reported to be associated with human reproductive cancers such as breast, prostate, and ovarian cancers [202–204]. In breast cancer, IGFBP-7 expression is inversely correlated with disease progression in breast cancer and can inhibit the growth of human breast cancer cells and xenograft tumors [205]. Further studies have shown that STAT5a inhibits the expression of IGFBP-7 by upregulating the expression of histone methyltransferase EZH2 and mediating H3K27 methylation in the IGFBP-7 promoter region, thereby promoting mitosis in breast cancer cells [206]. Additionally, IGFBP-7 inhibits tumor growth by binding to IGF-1 R to suppress IGF signaling and angiogenesis [82–85]. Recent studies have shown that IGFBP-7 can function as a chemokine, specifically recruiting CD8^+^ T cells to the tumor site while inhibiting Myeloid-derived suppressor cells (MDSCs) infiltration, thereby enhancing the tumor immune response [86]. Studies have also reported that IGFBP-7 can promote antigen presentation by dendritic cells (DCs), thereby recruiting CD4^+^ and CD8^+^ T cells [86]. In addition, IGFBP-7 secreted by hepatocytes inhibits IGF and NF-κB signaling in macrophages, preventing inflammation [86].

#### The tumor-promoting effect of IGFBP-7

In addition to its tumor suppressive function, when tumor cells express IGFBP-7 themselves, it can promote the anchoring-independent growth of malignant mesenchymal cells and epithelial cells with epithelial mesenchymal transformation phenotypes. And colon cancer cells can grow through tumor-stromal interactions [87]. The latest research reports that IGFBP-7 regulates the proliferation and migration of gastric cancer cells through the JAK/STAT pathway and is regulated by DNA and RNA methylation [88].

Plasma IGFBP-7 and IGFBP-7/IGF-1 ratio was positively correlated with age and obesity in postmenopausal women, which can increase the risk of cancer or premature death [207]. The serum levels of IGFBP-7 in colorectal cancer patients were significantly higher than those in normal controls, and serum IGFBP-7. The diagnostic accuracy as a biomarker has been significantly improved [208]. IGFBP-7 methylation status in oral tongue cancer and esophageal cancer was positively correlated with tumor invasion depth, locoregional recurrence, and cancer sequence [201, 209]. The above studies show that IGFBP-7 plays a key role as a tumor suppressor in a variety of cancers and can be used as a diagnostic biomarker and therapeutic target for these cancers.

## IGFBP-targeted immunotherapy

### IGFBP as cancer diagnostic biomarkers

Recent clinical studies indicate that high-risk patients with pancreatic ductal adenocarcinoma exhibit specific IGFBP expression patterns associated with aggressive tumor biology [14]. The IGFBP family genes are key regulators of pancreatic ductal adenocarcinoma progression and immune landscape remodeling, and they can serve as prognostic biomarkers and therapeutic targets [14]. Additionally, IGFBP signaling pathways are significantly activated in osteosarcoma and are associated with prognosis [210].

Clinical research has found that IGFBP-1, as a potential prognostic biomarker for lung adenocarcinoma, is positively correlated with poor prognosis in lung adenocarcinoma [211]. IGFBP-2 is highly expressed in endometrial cancer and glioma tissues, positively correlates with immunosuppressive cells and immune checkpoint molecules, and is associated with poor prognosis [122, 212]. In the Tam-01 phase III trial, IGFBP-3 can predict the efficacy of low-dose tamoxifen in the treatment of non-invasive breast cancer [213]. IGFBP-3 expression is reduced in liver cancer, melanoma, and esophageal squamous cell carcinoma tissues, and is associated with poor prognosis [214, 215]. IGFBP-4 is significantly downregulated in metastatic hepatocellular carcinoma and cervical cancer, and its expression is positively correlated with patient prognosis, serving as an independent risk factor for prognosis [160, 216]. IGFBP-5 has higher expression levels in patients with breast cancer and bladder cancer, and is associated with lower survival rates of patients [217, 218]. Gene risk model analysis found that IGFBP-6 shows significant prognostic value in glioblastoma multiforme [219]. The level of IGFBP-7 in patients with breast cancer and multiple myeloma have prognostic value. IGFBP-7 levels are associated with an increased risk of distant metastasis and all-cause mortality, and can serve as an independent prognostic biomarker [220].

### IGFBP-targeted therapy

#### ICA-CUR

Studies have shown that the combination treatment of icaretin-curcumin (ICA-CUR) can induce autophagy and ferroptosis in prostate cancer cells and alter lipid metabolism [221]. Further studies have found that ICA-CUR can inhibit the progression of prostate cancer by affecting the metabolism of intestinal flora and affecting the IGFBP-2/STAT3/PD-L1 pathway of tumor cells, thereby activating CD8^+^ T cells and inhibiting the progression of prostate cancer [42].

#### Curcumin

Curcumin, a common food ingredient derived from the turmeric plant, is a powerful remedy against tumorigenesis [222]. Curcumin has been found to activate p38, Activated p38 is again passed through and Binding elements in the IGFBP-5 promoter interact to activate C/EBPα, thereby inhibiting the progression of oral cancer [223].

#### CDDP

Cisplatin (CDDP) is one of the frontline chemotherapy drugs for treating esophageal squamous cell carcinoma. CDDP resistance has become a major challenge in cancer treatment [224]. Studies have shown that the suppression of IGFBP-5 is one of the mechanisms by which esophageal squamous cell carcinoma cells acquire cisplatin resistance, and increasing IGFBP-5 expression levels can reverse the cisplatin-resistant phenotype [225]. This provides a new direction for reversing CDDP resistance in ESCC and potentially other types of cancer, and further research will be conducted in the future.

#### DES

IGFBP-6 has shown an emerging role as a mediator of airway inflammation and tumor suppressive activity in different lung tumors [226]. Diethylstilbestrol (DES) is a synthetic estrogen used to treat advanced human prostate cancer [227]. Studies have shown that DES can induce the expression of IGFBP-6 in the prostate cancer cell line PC-3, thereby inhibiting cell proliferation [228]. Therefore, IGFBP-6 may be involved in the direct effect of DES on prostate cancer.

Although the above - mentioned targeted therapeutic strategies exhibit promising application prospects, they are currently all at the preclinical stage, presenting a significant translational gap. Further research should be carried out in the future to evaluate the safety, efficacy, and clinical - stage potential of these drugs.

### Potential targeted therapy strategies

#### IGFBP-2/STAT3 and PD-L1

In tumors such as pancreatic ductal adenocarcinoma, IGFBP-2 promotes tumor progression by activating the STAT3 pathway and inducing macrophages in the tumor microenvironment to polarize toward the M2 phenotype [119]. Further studies have found that in in vivo mouse tumor models, IGFBP-2 can also continuously activate the STAT3 signaling pathway, upregulating PD-L1 expression in M2-like macrophages, thereby suppressing the proliferation and activation of CD8^+^ T cells in a PD-L1-dependent manner, ultimately creating an immunosuppressive microenvironment [68]. This discovery reveals the critical sequential role of the IGFBP-2/STAT3/PD-L1 axis in tumor immune evasion, providing a clear theoretical basis for the development of novel combined immunotherapies. IGFBP-2/STAT3 inhibitors can block the polarization of M2 macrophages and the induced expression of PD-L1 from upstream, reversing the immunosuppressive microenvironment; whereas PD-L1 inhibitors can relieve the suppression of T cells by already expressed PD-L1. The combination of the two can achieve a dual upstream and downstream blockade of the immune evasion pathway.

#### Lactate and IGFBP-6

In the tumor microenvironment of glioblastoma, glioblastoma cells produce lactate, which promotes the expression of IGFBP-6. Subsequently, this leads to an upregulation of M2 markers and a downregulation of iNOS levels in microglia, ultimately facilitating tumor growth [81]. These findings suggest that lactate/IGFBP-6 metabolism has the ability to reshape the tumor microenvironment in glioblastoma and activate immune escape mechanisms. Therefore, targeting the inhibition of lactate production combined with IGFBP-6 signaling blockade may suppress the progression of glioblastoma.

#### IGFBP and VEGF

The IGFBP family plays a key role in tumor progression by regulating VEGF expression and angiogenesis. IGFBP-2 can enter the nucleus via its NLS, directly activating VEGF expression to promote angiogenesis and tumor growth [64]. On the other hand, IGFBP-5 exhibits tumor-suppressive effects in ovarian cancer, and its C-terminal can block angiogenesis by inhibiting the AKT/ERK and NF-κB-VEGF/MMP-9 signaling pathways [174]. These findings suggest that IGFBPs and VEGF signaling interact closely in tumor angiogenesis. Combined targeting of IGFBP (especially the pro-angiogenic IGFBP-2) and VEGF/VEGFR inhibitors may produce a synergistic effect in anti-tumor therapy by doubly inhibiting tumor angiogenic signaling, particularly for tumor types with high IGFBP expression and active angiogenesis. This strategy offers a new direction for improving the efficacy and overcoming resistance issues of existing anti-angiogenic treatments.

#### PAPP-A and PSA

Pregnancy-associated plasma protein-A (PAPP-A) is a zinc metalloproteinase that is highly expressed in most breast cancers [229]. As an important regulatory molecule in the IGF system, PAPP-A regulates the local bioavailability of IGF by proteolytically cleaving IGFBP-4 and IGFBP-5 [230, 231]. The cleavage of IGFBP-4 by PAPP-A exhibits unique IGF dependency; PAPP-A can only effectively cleave this substrate after IGF binds to IGFBP-4 [232]. PAPP-A enhances the biological activity of IGF by cleaving IGFBPs, thereby promoting IGF signaling [233]. In tumors, PAPP-A promotes tumor growth, invasion, and metastasis by enhancing the local effects of IGF [234, 235]. PSA, also known as kallikrein-related peptidase 3 (KLK3), is an IGFBP protease secreted by prostate epithelial cells [236]. Clinically, PSA serves as a core serum biomarker for prostate cancer screening and monitoring [237, 238]. In the regulation of the IGF system, PSA, as a key IGFBP protease, regulates the bioavailability of IGF by hydrolyzing IGFBP-3 and IGFBP-5, thereby influencing tumor cell proliferation and the progression of prostate cancer [239, 240]. The aforementioned studies suggest that IGFBP-specific proteases (such as PAPP-A and PSA) can serve as potential therapeutic targets for cancer treatment, and their targeted inhibition (e.g., through protease inhibitors, blocking cleavage, or restoring the intact function of IGFBPs) can facilitate the advancement of anti-tumor strategies.

### Main challenges

The same IGFBP may play opposite roles (such as promoting or inhibiting cancer) in different tumors or microenvironments, adding complexity to targeted therapy. Moreover, after blocking a single IGFBP or IGF signaling, tumors may compensate by upregulating other family members, leading to drug resistance. In terms of safety, the IGF system is intricately involved in normal metabolism and growth, so systemic inhibition may cause side effects such as metabolic disorders. A particularly pressing challenge is that the high circulating levels of IGFBPs, given their role in insulin regulation, pose a risk of systemic metabolic side effects, including glucose intolerance and insulin resistance. These off-target effects could limit the therapeutic window and necessitate careful monitoring during treatment.

Despite these challenges, precise interventions targeting specific IGFBP members, as well as combination strategies with immune checkpoints and other therapies, have emerged as a promising new area for overcoming resistance to tumor immunotherapy. Specifically, IGFBP-targeted drugs may synergize with existing immune checkpoint inhibitors (ICIs) such as anti-PD-1/PD-L1 agents by modulating the tumor microenvironment to enhance T-cell infiltration and activation, while simultaneously disrupting IGF-driven pro-survival signaling pathways that contribute to immune evasion and resistance to ICIs.

### Conclusion and perspectives

Although scientific research on the relationship between IGFBP and the immune system is very limited compared to endocrine and metabolic systems, IGFBP still has enormous clinical value in the immune system and will become an effective target for immunotherapy of various cancers. IGFBP and related signaling pathways are very important in the immune system. In addition to serving as the first or second messenger to regulate cell migration, differentiation, proliferation, and apoptosis, IGFBP also connects the crosstalk between immune cells. However, there are currently no reports on the expression and function of IGFBP in immune cells (such as T cells, B cells, and dendritic cells) in the tumor microenvironment, and further research in this area should be strengthened in the future. In addition, IGFBP serves as a bridge between immune cells and tumor cells, providing us with new drug targets. Therefore, the study of IGFBPs and their related bioactive molecules has great academic and clinical value.

## Data Availability

Not applicable.

## Abbreviation

IGFBP: Insulin-like growth factor-binding protein
IGF: insulin-like growth factor
IGF-1 R: IGF-1 receptor
IGF-2 R: IGF-2 receptor
IGFBP-rP: Insulin-like growth factor-binding protein-related protein
IR: Insulin receptor
Treg: Regulatory T cell
PPARα: Proliferator-activated receptor alpha
SALL1: Hepatic spart-like transcription factor 1
HBD: Heparin-binding domain
NLS: Nuclear localization sequence
ECM: Extracellular matrix
CA 125: Cancer Antigen 125
PSA: Prostate-specific antigen
PD-L1: Programmed death ligand 1
STAT3: Signal transducers and transcriptional activator 3
ICI: In clinical studies
ICA-CUR: Icaretin-curcumin
GRP78: Glucose regulatory protein
ICI 182,780: Fulvestrant
SAN: Sanguinarine
NNK: Nicotine-derived nitrosamine ketone
FTO: Obesity-associated protein
SEMA7A: Semaphorin7A
CIBP-4: C-terminal protein fragment
Tg1: Thyroglobulin type 1
CatB: Cathepsin B
MYBBP1A: MYB binding protein 1A
MSC-CM: Mesenchymal stem cell conditioned medium
C/EBPα: CCAAT/enhancer-binding protein α
RASSF1: Ras associated domain family member 1
TET: ten-eleven translocation
ROR1: Receptor tyrosine kinase 1
HER2: Human epidermal growth factor receptor 2
CREB: cAMP response element-binding protein
KD: Keloids
DES: Diethylstilbestrol
PHB2: Prohibitin-2
iNOS: Inducible nitric oxide synthase
MDSC: Myeloid-derived suppressor cell
DC: Dendritic cell
PAPP-A: Pregnancy-associated plasma protein-A
KLK3: Kallikrein-related peptidase 3
